# Adaptive Tracking of High-Maneuvering Targets Based on Multi-Feature Fusion Trajectory Clustering: LPI’s Purpose

**DOI:** 10.3390/s22134713

**Published:** 2022-06-22

**Authors:** Lei Wei, Jun Chen, Yi Ding, Fei Wang, Jianjiang Zhou

**Affiliations:** 1 School of Electronic and Information Engineering, Nanjing University of Information Science and Technology, Nanjing 210044, China; 201983270195@nuist.edu.cn (L.W.); 201983270191@nuist.edu.cn (Y.D.); 2 Key Laboratory of Radar Imaging and Microwave Photonics, Ministry of Education, Nanjing University of Aeronautics and Astronautics, Nanjing 210016, China; wangxiaoxian@nuaa.edu.cn (F.W.); zjjee@nuaa.edu.cn (J.Z.)

**Keywords:** low probability of intercept, multi-sensor management, multi-feature fusion, trajectory clustering, high-maneuvering target

## Abstract

Since the passive sensor has the property that it does not radiate signals, the use of passive sensors for target tracking is beneficial to improve the low probability of intercept (LPI) performance of the combat platform. However, for the high-maneuvering targets, its motion mode is unknown in advance, so the passive target tracking algorithm using a fixed motion model or interactive multi-model cannot match the actual motion mode of the maneuvering target. In order to solve the problem of low tracking accuracy caused by the unknown motion model of high-maneuvering targets, this paper firstly proposes a state transition matrix update-based extended Kalman filter (STMU-EKF) passive tracking algorithm. In this algorithm, the multi-feature fusion-based trajectory clustering is proposed to estimate the target state, and the state transition matrix is updated according to the estimated value of the motion model and the observation value of multi-station passive sensors. On this basis, considering that only using passive sensors for target tracking cannot often meet the requirements of high target tracking accuracy, this paper introduces active radar for indirect radiation and proposes a multi-sensor collaborative management model based on trajectory clustering. The model performs the optimal allocation of active radar and passive sensors by judging the accumulated errors of the eigenvalue of the error covariance matrix and makes the decision to update the state transition matrix according to the magnitude of the fluctuation parameter of the error difference between the prediction value and the observation value. The simulation results verify that the proposed multi-sensor collaborative target tracking algorithm can effectively improve the high-maneuvering target tracking accuracy to satisfy the radar’s LPI performance.

## 1. Introduction

In modern warfare, when the radar detects and tracks the target, its radiation signal is easily intercepted by the intercepting receiver, which seriously threatens the survivability of the radar on the battlefield. Low probability of intercept (LPI) has become one of the essential properties of radar. Only with the LPI performance can radar survive in the harsh and fierce electronic warfare and exert its tactical and technical performance [1,2]. Therefore, the research on LPI radar has become one of the hot issues in modern radar research, attracting more and more scholars.

Common radar low intercept probability realization methods include low intercept probability waveform design, low sidelobe antenna design, and low radiation energy control. Among them, low radiation energy control is the most direct and effective method to achieve the LPI performance of the radar. For radar low radiation energy control, a wealth of research results has been achieved. Radar low radiation energy control mainly includes radar low radiation power control and radar low radiation time control. In terms of airborne radar radiation power control, Godrich et al. proposed a multiple-input multiple-output (MIMO) radar radiated power control algorithm that satisfies the radar target positioning accuracy under the Cramér–Rao bound of distributed MIMO radar target state estimation [3]. For multi-target tracking, Chavali et al. proposed a joint optimization algorithm for MIMO radar transmit antenna selection and radiation power [4]. Shi et al. proposed that on the premise of meeting the requirements of target tracking performance, the power distribution of the networked radar is carried out by minimizing the total transmit power of the networked radar so as to achieve the radio frequency stealth performance of the networked radar [5]. Xie et al. optimized the radar node selection and power allocation in the multi-target tracking process of the networked radar [6]. Han et al. studied the power allocation problem of opportunistic array radar using the fuzzy variable representation method [7]. Ghoreishian et al. established an RF stealth power allocation model for distributed MIMO radar under two transmit waveforms, orthogonal frequency diversity, and phase encoding, respectively, for extended targets [8]. Based on the application of distributed MIMO radar target detection, Jebali et al. proposed a power allocation method that jointly optimizes the total transmit power and intercept probability [9]. In terms of airborne radar radiation time control, Wang et al. studied the joint assignment of beam pointing and dwell time in the multi-target tracking process of phased array radar [10]. Ghazal M et al. studied infrared sensors and electronic support measures to assist airborne radar in target tracking, which can effectively reduce the number of external radiations of airborne radar [11]. Xiong et al. also proposed to further increase the silence time of the airborne radar through the cross-location method of multiple infrared sensors [12]. Narykov et al. studied radar time resource management under the joint optimization of sensor selection and dwell time for networked radar [13]. For MIMO radar, Zhao et al. proposed that in MIMO radar search mode, the radio frequency stealth performance of MIMO radar can be achieved by adaptively controlling beam dwell time, signal duty cycle, and search frame period [14]. Shaghaghi et al. introduced machine learning into the multi-channel, multi-function radar resource management problem, solved the optimal solution of the task scheduling problem by using the branch and bound algorithm, and used machine learning to reduce the computational complexity and maximize the utilization of time and other resources [15]. Han et al. proposed a joint adaptive sampling interval and power allocation (JASIPA) scheme based on opportunistic programming constraint (OCP) [16]. However, most of the existing low radiation energy control methods are designed for conventional moving targets, and there are few studies on high-maneuvering targets. This paper focuses on the radar low radiation time control of high-maneuvering targets. The key is to improve the tracking accuracy of passive sensors for high-maneuvering targets. In the process of low radiation energy tracking for the target, this paper will realize the adaptive update of the target motion state model based on the target motion trajectory clustering algorithm.

In terms of target trajectory clustering technology, combining trajectory mapping and clustering methods, Li et al. proposed an improved density-based applied spatial clustering algorithm with noise (DBSCAN) to cluster spatial points to obtain optimal clusters [17]. Yu et al. proposed an efficient trajectory dimensionality reduction method and a DBSCAN hyperparameter initialization method [18]. In order to achieve adaptive parameter calibration and reduce the workload of trajectory clustering, Mao et al. proposed an adaptive trajectory clustering method based on grid and density [19]. Aiming at the limitation that trajectory clustering is often sensitive to undesired outliers, Li et al. proposed a multi-step trajectory clustering method for robust AIS trajectory clustering [20]. To address the computational complexity of the DENCLUE algorithm, Mariam et al. conducted an empirical evaluation of using the DENCLUE algorithm to discover clusters of arbitrary shapes [21]. In the application of AIS trajectory separation, Lei et al. used the OPTICS clustering method based on spatiotemporal distance [22]. Aiming at the problems of difficult parameter setting, high time complexity, poor noise recognition, and weak clustering ability for data sets with uneven density in most density-based clustering algorithms, Tang et al. proposed an improved OPTICS algorithm to overcome the weakness of most algorithms for clustering in data sets with uneven density [23]. However, most of the common trajectory clustering algorithms use one single feature for clustering and cannot make accurate judgments on maneuvering targets with similar motion patterns. Aiming at the single eigenvalue problem of the traditional OPTICS algorithm, this paper proposes a trajectory clustering algorithm based on multi-feature fusion. On this basis, the motion state estimation is performed, and the state transition matrix is updated according to the observation values of multi-station passive sensors.

It can be seen from the current research status of low radiation energy control and trajectory clustering that most of the existing low radiation energy control methods do not take high-maneuvering targets into account. Moreover, common trajectory clustering algorithms use scant features and cannot make accurate judgments on maneuvering targets that have similar motion patterns. Aiming at these issues, we conduct research on them. In this paper, a multi-feature fusion-based trajectory clustering algorithm is proposed to improve the clustering accuracy. On this basis, the STMU-EKF algorithm is proposed to solve the problem of low tracking accuracy caused by the unknown motion model of high-maneuvering targets. Considering that only using passive sensors for target tracking cannot often meet the requirements of high target tracking accuracy, this paper also introduces active radar for indirect radiation and proposes a multi-sensor collaborative management model based on trajectory clustering.

The rest of this paper is organized as follows. Section 2: A trajectory clustering algorithm based on multi-feature fusion is proposed. Section 3: On the basis of the multi-feature fusion-based trajectory clustering, a passive target tracking algorithm for high-maneuvering targets is proposed. Section 4: To satisfy the LPI performance of radar and the target tracking accuracy, a multi-sensor collaborative management model based on trajectory clustering is proposed. Section 5: Put the above algorithms into simulation. Section 6: Conclude this paper.

## 2. Trajectory Clustering Algorithm Based on Multi-Feature Fusion

### 2.1. Trajectory Feature Description

The movement trajectory of a maneuvering target is essentially a mapping from time to space, and the trajectory contains the relevant target information in time, space, and its own properties. In a specific environment, by tracking the maneuvering target, a series of centroid points can be obtained, and the target motion trajectory can be obtained by connecting the above centroid points in a time sequence. Assuming that the coordinates in the two-dimensional space are $\left(x_{k},\;y_{k}\right)$ and the current timestamp is $t_{k}$, the target trajectory can be expressed by

(1) $$T=\left\{x_{k},y_{k},t_{k},k=1,2,\ldots ,N\right\}.$$

During the movement process of a maneuvering target, when the trajectory model changes, the trajectory between different frames is different, and the spatial trajectory distribution of the specific model of the maneuvering target has certain characteristics. As one of the bases of the trajectory model, the maneuvering target’s inter-frame trajectory mean M can be defined as a position trajectory feature and can be written as

(2) $$M=\frac{1}{N}\overset{N}{\underset{k=1}{\sum }}\left(x_{k},y_{k}\right).$$

The spatial information of the target trajectory T also includes the information $L_{k}$ of its trajectory length and forward direction, which can be defined as

(3) $$L_{k}=\left(x_{k}-x_{k-1},y_{k}-y_{k-1}\right),$$

where $x_{k}$ and $y_{k}$ represent the trajectory vectors in the horizontal and vertical directions at time $t_{k}$, respectively. The trajectory type can be divided according to the size of $L_{k}$, which can be defined as another trajectory feature. For example, the larger the absolute value ${|L}_{k}|$ of the information $L_{k}$ is, the higher the probability of linear motion is, and according to the positive and negative of $L_{k}$, the direction of trajectory movement can be judged. For example, the trajectory with a negative sign is generally moving in the opposite direction.

However, for Equation (3), only the rough movement direction of the target can be distinguished, that is, forward or backward. If you want to describe the movement direction of the maneuvering target more accurately, you need to divide the trajectory between frames. The trajectory information $L_{k}$ should be further expanded into the angle information and the speed information.

For the trajectory angle information, we can set a certain angle threshold. When the trajectory angle exceeds the threshold, it will be marked as the corresponding feature point. According to the distribution of the feature points, the trajectory angle $\theta_{k}$, which can be defined as an instantaneous angle trajectory feature, can be defined as

(4) $$\theta_{k}=\arctan(\frac{y_{k}-y_{k-1}}{x_{k}-x_{k-1}})+\frac{\pi }{2}+n\pi .$$

For the trajectory speed information, we can also use the size of the target movement speed as the target clustering feature, which can be further divided into instantaneous speed $v_{k}$ and average speed $\bar{v}$. They can be written respectively as

(5) $$v_{k}=\sqrt{{\left(x_{k}-x_{k-1}\right)}^{2}+{\left(y_{k}-y_{k-1}\right)}^{2}},$$

(6) $$\bar{v}=\frac{1}{N-1}\sum_{k=1}^{N-1}v_{k},$$

where the successive time intervals are the same.

When the instantaneous speed $v_{k}$ is too high, the probability of making a turning motion at this moment is small. When the average speed $\bar{v}$ is too high, the probability of making a linear motion in the whole process is large.

### 2.2. Trajectory Clustering Algorithm Based on Multi-Feature Fusion

Common trajectory clustering algorithms include hierarchical-based clustering, density-based clustering, partition-based clustering, grid-based clustering, and model-based clustering. Among them, the density-based clustering algorithm, including the OPTICS algorithm, the DBSCAN algorithm, and the DENCLUE algorithm, can obtain more clustering accuracy by searching for different clusters and requires fewer input parameters. Compared with other density-based clustering algorithms, OPTICS is an improved density clustering algorithm. It shares many common concepts with the DBSCAN algorithm, such as core objects, density of direct, density connection, etc. However, it overcomes the shortcomings of the DBSCAN algorithm’s sensitivity to initial setting parameters and poor adaptability to data sets with different densities and requires fewer input parameters than the DENCLUE algorithm, which is suitable for trajectory recognition in the process of high-maneuvering target tracking.

Based on the DBSCAN algorithm, the OPTICS algorithm introduces two important definitions, namely core distance d(x) and reachable distance r(x,y).

Assuming that the sample x∈X, its $R_{\in }$ neighborhood contains the number of sub-sample sets in the sample set *X* whose distance from *x* is not greater than $R_{\in }$ is $N_{R_{\in }}\left(x\right)$, the input parameters are $(R_{\in },\;\mathrm{MinPts})$, where $R_{\in }$ is the radius, MinPts is the minimum number of points. The value d(x) of the minimum neighborhood radius of the sample core point, which is called the core distance of x, is obtained according to the given parameters that is

(7) $$d\left(x\right)=d\left(x,N_{R_{\in }}^{MinPts}\left(x\right)\right),\left|N_{R_{\in }}\left(x\right)\right|\geq MinPts,$$

where $N_{R_{\in }}^{MinPts}\left(x\right)$ is the *i*th node adjacent to node MinPts in set $N_{R_{\in }}\left(x\right)$.

The reachable distance r(x,y) represents the minimum neighborhood radius that can be directly density-reachable from the core point x, which is

(8) $$r\left(x,y\right)=\min\{R_{\in }:y\in N_{R_{\in }}\left(x\right)\}.$$

Traditional OPTICS clustering algorithms often use a single eigenvalue for clustering, for example, using the motion vector between two directly adjacent frames as a single eigenvalue for clustering. However, a single eigenvalue only reflects the characteristics of a certain aspect of the trajectory. If a single eigenvalue is used for clustering, an accurate judgment cannot be made for maneuvering targets with similar motion patterns.

In order to further improve the model matching accuracy, this paper uses four spatial features: the maneuvering target’s inter-frame trajectory mean M, trajectory angle $\theta_{k}$, instantaneous speed $v_{k}$ and average speed $\bar{v}$ for trajectory clustering, which corresponds to different feature spaces. For the fusion clustering, the clustering results from different feature spaces need to be associated. Then the relationship between the obtained fusion clustering results and the remaining unprocessed trajectories can be established based on the conditional probability of them. Finally, the motion model of the trajectory can be obtained. Since the multi-feature fusion-based clustering is performed after the clustering of each feature space, the dimensionality problem caused by fusion before clustering in traditional algorithms can be avoided.

Assume that the feature space sample set is ${D=\{F}_{1},\ldots ,F_{n}\}$, where n is the number of feature spaces. If each feature space sample $F_{n}$ in D is clustered separately, the sample spaces will obtain different numbers of clusters ${\{N}_{i}\left|i=1,\ldots ,n\right\}$. After the fusion clustering is performed, the clustering parameters need to contain the clustering results of all the feature spaces. Therefore, the number $N_{\max}$ of clusters after fusion clustering is the maximum value in $N_{i}$, that is, $N_{\max}{=\max\{N}_{1},\ldots ,N_{n}\}$. The clustering result after fusion can be expressed as ${\{C}_{\max}^{l}\left|l=1,\ldots ,n\right\}$.

After the clustering results of each feature space are obtained, different feature spaces need to be associated. Assuming that the two feature spaces are $F_{a}$ and $F_{b}$, respectively, the lth clustering result of $F_{b}$ is $E_{b}^{l}$, and the cluster of the most overlapping elements with $E_{b}^{l}$ in the clustering result of $F_{a}$ is $E_{a}^{\beta }$. Then the clustering result can be updated to

(9) $$E_{max}^{l}=C_{max}^{l}+E_{a}^{\beta }\left(l=1,\ldots ,N_{max}\right).$$

In order to correlate with the remaining unprocessed trajectories, a probabilistic mapping relationship between each trajectory and the clustering of existing trajectories needs to be calculated. Assuming that the trajectories that have not yet been clustered are G, the standard deviation of $E_{\max}^{l}$ is $\delta_{\max}^{l}$, and the mean value is $\mu_{i}$, the conditional probability between each trajectory and the corresponding fusion clustering result is

(10) $$P(G|C_{max}^{l})=\frac{1}{n}\sum_{i=1}^{n}\frac{1}{\sqrt{2\pi }\delta_{max}^{l}}\exp(\frac{x_{i}-\mu_{i}}{\delta_{max}^{l}}).$$

From the obtained conditional probabilities, we can then make decisions on the assignment of the remaining trajectories. When ${P(G|E}_{\max}^{l}{)>P(G|E}_{b}^{l})$, the corresponding trajectory G will establish a connection with $E_{\max}^{l}$. When all remaining trajectories have been assigned, the fusion clustering algorithm ends. The steps of the above algorithm are shown in Algorithm 1:

**Algorithm 1.** The steps of trajectory clustering algorithm based on multi-feature fusion.**Step1.** Count the required number of clusters after trajectory fusion $N_{\max}$. **Step2.** Calculate the clustering structure $E_{\max}^{l}$ of this target movement, and complete the fusion trajectory clustering for each individual feature space. **Step3.** Assign the trajectories that satisfy the clustering conditions to the corresponding clusters by calculating the conditional probability ${P(G|C}_{\max}^{k}$ and making judgments. **Step4.** Obtain the approximate model of the target motion trajectory according to the result of trajectory clustering after multi-feature fusion.

## 3. Passive Tracking Algorithm for High-Maneuvering Targets Based on Adaptive Update of Target Motion State Model

As a common passive sensor target tracking algorithm, the extended Kalman filter (EKF) algorithm is often used in the condition where the target trajectory is nonlinear. The principle is to truncate the nonlinear state equation f(x) and the observation equation h(x) according to the target filter value by the first-order Taylor series so as to obtain a linearized system model. However, the state transition matrix of the conventional EKF algorithm has the singularity property and will keep unchanged in the process of high-maneuvering target tracking, causing a large error in the filtering results. IMM-EKF is based on the probability of different motion models, which can improve the tracking accuracy in maneuvering target tracking to some extent. For high-maneuvering targets, IMM-EKF is difficult to obtain the motion model probability in advance, so it cannot improve its tracking accuracy effectively. In order to solve this problem, this section introduces the multi-feature fusion-based trajectory clustering algorithm proposed in Section 2 and designs a state transition matrix update-based EKF (STMU-EKF) passive tracking algorithm for high-maneuvering targets.

**Figure 1:**
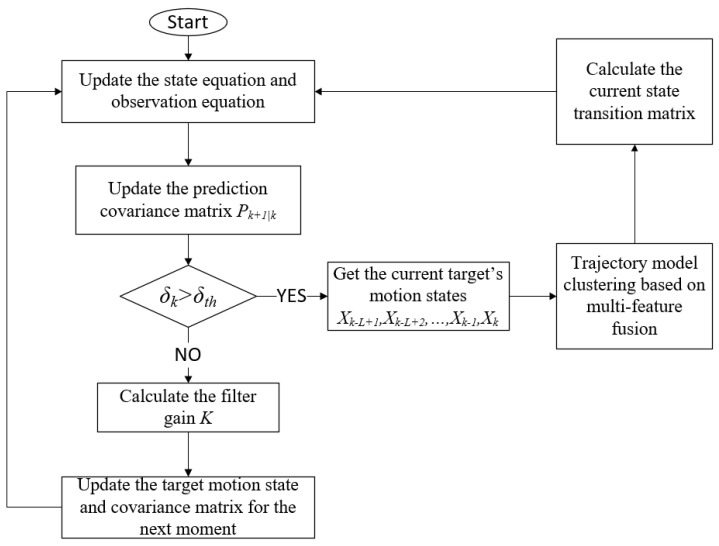
Flowchart of the STMU−EKF algorithm.

The proposed STMU-EKF algorithm can be divided into three main steps, which are the prediction of the current target (also called ‘time update’), state correction (also called ‘state transition matrix update’), and parameter correction (also called ‘measurement update’). The flowchart of the proposed STMU-EKF algorithm is shown in Figure 1.

### 3.1. Time Update

Assuming that the current target motion state is ${\hat{X}}_{k}$, the covariance matrix of the target state vector is $P_{k}$, and the measurement noise is $v_{k}$. The nonlinear state equation is f(x), and the nonlinear observation equation is h(x), then the Jacobi matrices of these two nonlinear equations are calculated as $F_{k}$ and $H_{k}$, which is $F_{k}=\frac{\partial f(X_{K-1})}{\partial X_{K-1}}{|}_{X_{K-1}={\hat{X}}_{k-1|k-1}}$ and $H_{k}=\frac{\partial f(X_{K})}{\partial X_{K}}{|}_{X_{K}={\hat{X}}_{k|k-1}}$.

The state equation is

(11) $${\hat{X}}_{k+1|k}=f\left({\hat{X}}_{k|k}\right)=F_{k+1|k}{\hat{X}}_{k|k},$$

where $F_{k+1|k}$ represents the state transition matrix.

The observation equation is

(12) $${\hat{Z}}_{k+1|k}=H_{k+1|k}{\hat{X}}_{k+1|k}+v_{k}.$$

And the prediction covariance matrix is

(13) $$P_{k+1|k}=F_{k+1|k}P_{k|k}F_{k+1|k}^{T}+Q_{k+1},$$

where $Q_{k+1}$ represents the Gaussian covariance matrix in the target prediction process.

### 3.2. State Transition Matrix Update

Through Equations (11)–(13), we have completed the time update of the target, and then we need to update the state transition matrix by decision.

The state transition matrix is determined by the target state model, and the target motion state can be estimated by multi-feature fusion trajectory clustering according to the observation values of the target state, which can be obtained by a multi-station passive positioning system. The difference time of arrival (DTOA) algorithm, which is one kind of multi-station passive positioning algorithm, can obtain the target state information. Since it does not radiate electromagnetic signals to the outside, it will not be intercepted by the interceptor. Therefore, this paper applies DTOA to help with the state transition matrix update of the STMU-EKF algorithm, extracts the features of the parameters obtained by the observation value of DTOA, and carries on the multi-feature fusion-based trajectory clustering according to the extracted features.

**Figure 2:**
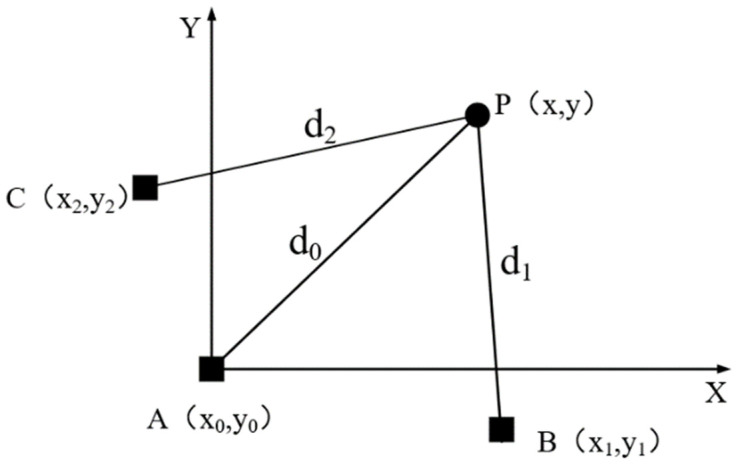
Three station time difference positioning model.

DTOA uses the time difference of electromagnetic signals to reach different stations to construct the hyperboloid of the target position and calculates the intersection of different hyperboloids to obtain the target position. The model of time difference multi-station passive positioning is shown in Figure 2.

In Figure 2, point P represents the radiation source, and points A, B, and C represent the three passive sensors that track the target P. Assuming that at time k, the coordinate of the radiation source is $P(x_{k},y_{k})$, and the position coordinates of the three passive sensors are ${A(x}_{0}{,y}_{0})$, ${B(x}_{1}{,y}_{1})$, ${C(x}_{2}{,y}_{2})$. The distances of the radiation source to the three passive sensors are $d_{0}$, $d_{1}$ and $d_{2}$, respectively, which have the mathematical relationship with the target position as

(14) $$\{\begin{array}{c} d_{0}{}^{2}={\left(x_{k}-x_{0}\right)}^{2}+{\left(y_{k}-y_{0}\right)}^{2}\\ \begin{array}{c} d_{1}{}^{2}={\left(x_{k}-x_{1}\right)}^{2}+{\left(y_{k}-y_{1}\right)}^{2}\\ d_{2}{}^{2}={\left(x_{k}-x_{2}\right)}^{2}+{\left(y_{k}-y_{2}\right)}^{2} \end{array} \end{array}.$$

Assuming that the position of passive sensor A is the main positioning and tracking station, and the positions of passive sensors B and C are auxiliary positioning and tracking stations, c represents the propagation speed of electromagnetic waves in the air, and the time difference between the time the signal reaches each auxiliary station and the time it reaches the main station is

(15) $$\Delta t_{0-i}=\frac{d_{0}-d_{i}}{c}.$$

Performing calculations on Equations (14) and (15), the corresponding hyperbolic equations are obtained as follows

(16) $$\{\begin{array}{c} \sqrt{{\left(x_{k}-x_{0}\right)}^{2}+{\left(y_{k}-y_{0}\right)}^{2}}-\sqrt{{\left(x_{k}-x_{1}\right)}^{2}+{\left(y_{k}-y_{1}\right)}^{2}}=c\Delta t_{0-1}\;\\ \sqrt{{\left(x_{k}-x_{0}\right)}^{2}+{\left(y_{k}-y_{0}\right)}^{2}}-\sqrt{{\left(x_{k}-x_{2}\right)}^{2}+{\left(y_{k}-y_{2}\right)}^{2}}=c\Delta t_{0-2} \end{array}.$$

According to Equation (16), the observation value of the current model is $\Delta t_{0-i}$, and the difference between the distances of the measured target reaching the main station and the auxiliary station is $d_{0}-d_{i}=c\Delta t_{0-i}$, i=1,2. Taking the distance difference $c\Delta t_{0-i}$ calculated here as the observation value $Z_{k}^{\mathrm{DTOA}}$ of DTOA, which is

(17) $$Z_{k}^{DTOA}=\left[Z_{\Delta d_{1}},Z_{\Delta d_{2}},Z_{\Delta d_{3}}\right],$$

where $\Delta d_{i}{=d}_{0}-d_{i}$, and $Z_{\Delta d_{i}}$ is the observation value of $\Delta d_{i}$. The observation equation can be obtained by Equation (17), which is

(18) $${\hat{Z}}_{k+1|k}^{DTOA}=H_{k+1|k}^{DTOA}{\hat{X}}_{k+1|k}+v_{k},$$

where $H_{k+1|k}^{DTOA}{\hat{X}}_{k+1|k}={\left[d_{0}-d_{1},d_{0}-d_{2},d_{0}-d_{3}\right]}^{T}$. According to Equations (17) and (18), the features required by the clustering algorithm can be obtained so as to estimate the target state model and update the state transition matrix.

For the decision to update the state transition matrix, this paper introduces the fluctuation parameter $\delta_{k}$, which reflects the accuracy of the filtering algorithm. It is the difference in the error between the prediction value ${\hat{X}}_{k}$ and the observation value $X_{k}$ of the filtering algorithm in two adjacent intervals, which can be expressed as

(19) $$\delta_{k}=\|{\hat{X}}_{k}-X_{k}{\|}_{1}-\|{\hat{X}}_{k-1}-X_{k-1}{\|}_{1},$$

where ${\left||\cdot |\right|}_{1}$ represents vector 1 norm. When the target motion state model is unchanged, the $\delta_{k}$ in the two adjacent time intervals is smaller, and when the target motion state model changes because the state transition matrix no longer adapts to the current motion model, the $\delta_{k}$ is larger. 

Set the preset error threshold as $\delta_{\mathrm{th}}$. When $\delta_{k}$ is not greater than the preset threshold $\delta_{th}$, keep the state transition matrix $F_{k+1|k}$ unchanged; otherwise, if $\delta_{k}$ > $\delta_{\mathrm{th}}$, then update the state transition matrix $F_{k+1|k}$. By using the multi-feature fusion-based trajectory clustering algorithm proposed in Section 2.2 for target trajectory clustering, the approximate motion trajectory model of the target can be obtained. The current state transition matrix F of the target can be inferred by the trajectory model. If the target moves in a straight line at a uniform speed, the state transition matrix is

(20) $$F=\left[\begin{array}{cccc} 1 & T & 0 & 0\\ 0 & 1 & 0 & 0\\ 0 & 0 & 1 & T\\ 0 & 0 & 0 & 1 \end{array}\right],$$

where T represents the current sampling interval. 

Similarly, if the target performs a coordinated turning motion, the state transition matrix is

(21) $$F=\left[\begin{array}{cccc} 1 & \sin\omega /\omega & 0 & \left(\cos\omega -1\right)/\omega \\ 0 & \cos\omega & 0 & -\sin\omega \\ 0 & \left(1-\cos\omega \right)/\omega & 1 & \sin\omega /\omega \\ 0 & \sin\omega & 0 & \cos\omega \end{array}\right],$$

where ω represents the current angular velocity of the target movement.

In the process of target tracking, many motion parameters can be obtained, such as target radial distance $\rho_{k}$, target azimuth angle $\theta_{k},$ and target pitch angle $\varphi_{k}$. Substituting the current sampling interval T into Equation (20), we can obtain the current state transition matrix $F_{k}$ of the uniform linear motion model. According to the target pitch angle $\varphi_{k}$ and the current sampling interval T obtained by the sensor, the angular velocity ω(k+1|k) between target frames can be calculated, and the angular velocity $\omega_{k}$ can be calculated as

(22) $$\omega_{k}=\frac{1}{L}\sum_{l=-\left(L-1\right)}^{0}\omega (k+l+1|k+l)=\frac{1}{L}\sum_{l=-\left(L-1\right)}^{0}\frac{\varphi_{k+l+1}-\varphi_{k}}{T}.$$

Substituting $\omega_{k}$ into Equation (21), we can obtain the current state transition matrix $F_{k}$ of the coordinated turning motion model.

### 3.3. Measurement Update

Substituting $F_{k}$ into Equation (13), we can obtain a new covariance matrix, which is

(23) $$P_{k+1|k}=F_{k}P_{k|k}{\left(F_{k}\right)}^{T}+Q_{k+1}.$$

After the decision update of the state transition matrix, the filter parameters need to be updated. Among them, the filter gain K represents the degree of the uncertainty of the result after data fusion, and its calculation equation is

(24) $$K_{k+1|k}=P_{k+1|k}{\left(H_{k+1}^{DTOA}\right)}^{T}{\left[H_{k+1}^{DTOA}P_{k+1|k}{\left(H_{k+1}^{DTOA}\right)}^{T}+R_{k}\right]}^{-1},$$

where $R_{k}$ represents the covariance matrix of the measurement error. According to the obtained filter gain K, the current state can be estimated by ${\hat{X}}_{k+1|k+1}$, which is,

(25) $${\hat{X}}_{k+1|k+1}={\hat{X}}_{k+1|k}+K_{k+1}\left[Z_{k+1}^{DTOA}-H_{k+1}^{DTOA}{\hat{X}}_{k+1|k}\right].$$

The covariance matrix $P_{k+1}$ of the current target state vector can be updated as

(26) $$P_{k+1}=P_{k+1|k}-K_{k+1}H_{k+1}^{DTOA}P_{k+1|k}.$$

## 4. Multi-Sensor Collaborative Management Model Based on Trajectory Clustering

**Figure 3:**
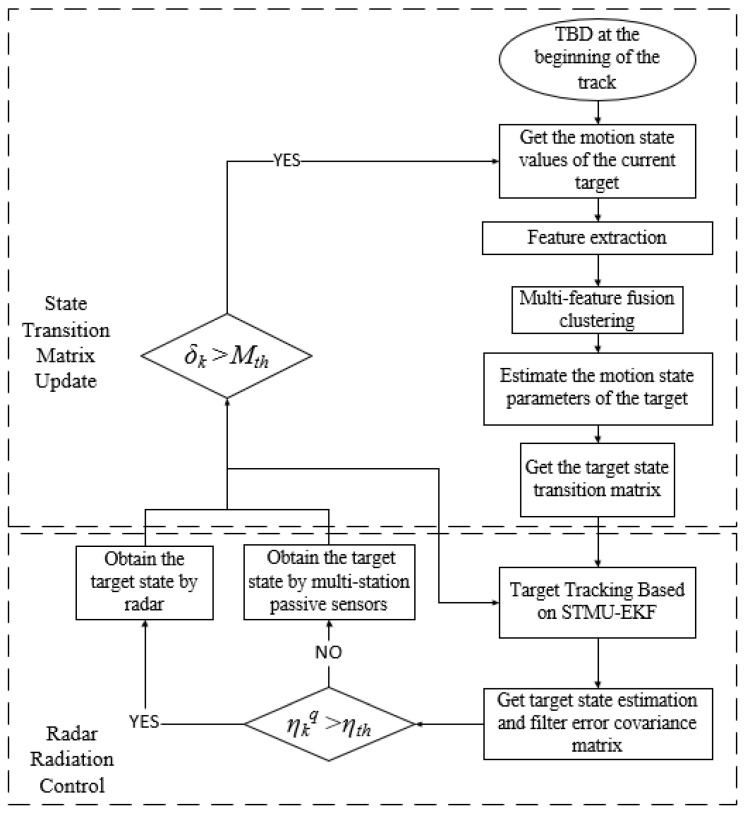
Multi-sensor collaborative management model based on trajectory clustering.

Considering that the passive sensors have a low positioning accuracy for high-maneuvering targets, the STMU-EKF algorithm proposed in Section 3 has an unsatisfactory tracking error by only using the passive sensors, and with the iteration of the filtering algorithm, the target tracking effect will become worse and worse. To address this problem, this section proposes a multi-sensor collaborative management model based on trajectory clustering. In this model, the trajectory parameters are corrected through radar radiation. Since the radar’s radiation signal is easily intercepted by the intercepting receiver, this paper applies indirect radar radiation for the target state transition matrix update, thus achieving the LPI tracking of high-maneuvering targets. The multi-sensor collaborative management model based on trajectory clustering is shown in Figure 3.

The initial target motion state $X_{0}$ and its state transition matrix F can be obtained by TBD at the beginning of the track. In the two-dimensional rectangular coordinate system, the maneuvering target motion state $X_{k}$ can be expressed as

(27) $$X_{k+1}=FX_{k}+W_{k},$$

where $W_{k}$ represents Gaussian white noise with a mean of 0.

And the observation value $Z_{k}^{j}$, which is observed from radar when j=1, while observed from the multi-station passive sensors when j=2, can be expressed as

(28) $$Z_{k+1}^{j}=HX_{k}+V_{k},$$

where $V_{k}$ represents Gaussian white noise with a mean of 0.

When the radar is used as the observation sensor for target tracking, that is, when j=1, the observation value $Z_{k}^{1}$ is

(29) $$Z_{k}^{1}=\left[\rho \left(k\right),\theta \left(k\right),\varphi \left(k\right)\right],$$

where ρ(k) is the distance of the target measured by radar, θ(k) is the target azimuth, and φ(k) is the target pitch angle. If the multi-station passive sensor is used as the observation sensor and the DTOA is used for target tracking, that is, when j=2, the observation value $Z_{k}^{2}$ is

(30) $$Z_{k}^{2}=\left[Z_{\Delta d_{1}},Z_{\Delta d_{2}},Z_{\Delta d_{3}}\right].$$

As shown in Figure 3, the sensor management model is mainly divided into two parts, which are the state transition matrix update and the radar radiation control.

### 4.1. State Transition Matrix Update

Firstly, the state transition matrix is updated. At the beginning of the track, the target motion state is obtained by the TBD algorithm, and the features are extracted from the target motion state values $X_{k-L+1}{\sim X}_{k}$. In order to improve the accuracy of trajectory model recognition, the four spatial features of the maneuvering target, namely, the inter-frame trajectory mean M, the instantaneous trajectory angle $\theta_{k}$, the instantaneous speed $v_{k},$ and the average speed $\bar{v}$ are used for multi-feature fusion.

After the feature extraction, the current target motion state is clustered by using the multi-feature fusion-based clustering algorithm described in Section 2.2 in this paper, and the current trajectory model of the target is obtained. According to the trajectory model, the target current state transition matrix F can be inferred.

According to the observation value $Z_{k}^{j}$ obtained by radar observation and the state transition matrix F obtained by trajectory clustering, the target tracking algorithm can obtain the prediction value ${\hat{X}}_{k+1|k+1}$ and the prediction covariance $P_{k+1}$, as shown in Equations (25) and (26).

When using the multi-sensor collaborative management model for target tracking, we also use the fluctuation parameter $\delta_{k}$ proposed in Section 3 as the basis for updating the state transition matrix. Assuming that the threshold is $M_{\mathrm{th}}$ in the multi-sensor collaborative management model, when $\delta_{k}$ > $M_{\mathrm{th}}$, it means that the target tracking error has increased sharply, and the target trajectory has changed abruptly. At this time, the motion states of the current target are updated again, and the features are extracted for fusion and clustering, and then the target state transition matrix can be obtained by estimating the target motion state parameters. Finally, the updated target state transition matrix is given to the target tracking algorithm for the next round of tracking. 

### 4.2. Radar Radiation Control

In the radar radiation control part, in order to measure the accuracy of the current multi-sensor collaborative management model so as to carry out the optimal allocation of sensors, we take the accumulated errors of the eigenvalue of the error covariance matrix $\eta_{k}^{q}$ as the basis for sensor decision making, which refers to the stacking value of the difference between the traces of the prediction covariance matrices in adjacent intervals from time q to time k, which is

(31) $$\begin{array}{ll} \eta_{k}^{q}= & \overset{k}{\underset{i=q}{\sum }}\left(T_{r}(P_{i+1}\right)-T_{r}(P_{i}))\\ & =\sum_{i=q}^{k}[T_{r}\left(P_{i+1|i}^{m}-K_{i+1}H_{i+1}^{DTOA}P_{i+1|i}^{m}\right)-T_{r}\left(P_{i|i-1}^{m}-K_{i}H_{i}^{DTOA}P_{i|i-1}^{m}\right)], \end{array}$$

where $T_{r}$ refers to the trace of the matrix.

The preset threshold of $\eta_{k}^{q}$ is $\eta_{\mathrm{th}}$, and $\eta_{k}^{q}$ of the target tracking algorithm is compared with the preset threshold $\eta_{\mathrm{th}}$. If $\eta_{k}^{q}\leq \eta_{\mathrm{th}}$, no radar radiation is performed, the passive DTOA is used for target tracking, and the obtained parameters are used as the input parameters of the target tracking algorithm for the next filter tracking; if $\eta_{k}^{q}>\eta_{\mathrm{th}}$, it means that the error of the target tracking algorithm is too large, and the passive DTOA with poor accuracy can no longer be used for tracking. At this time, the radar radiation is turned on, and the observation value obtained by the radar is used as the observation value required by the target tracking algorithm at the next moment to carry out the next round of target tracking. By repeating the above steps, we can achieve LPI tracking.

## 5. Simulations and Performance Analysis

### 5.1. Simulation Parameters

Without loss of generality, the tracking process is implemented in the two-dimensional X-Y coordinate system. In the coordinate system, the initial position of the target is (30 km, 100 km), and the initial speed is (150 m/s, 260 m/s). The measurement standard deviation error of the radar in the distance is 20 m, and the measurement standard deviation error in azimuth and pitch angle is 0.1°; the measurement standard deviation error of azimuth and pitch angle of the passive sensor is 0.4°. The minimum measurement interval for radar and passive sensors is 3 s, and the number of sampling points is 85. The initial motion model of the target is a uniform linear model, which suddenly changes to a collaborative turning model at 120 s and continues to move with this model until the measurement ends at 255 s.

### 5.2. Trajectory Clustering

**Figure 4:**
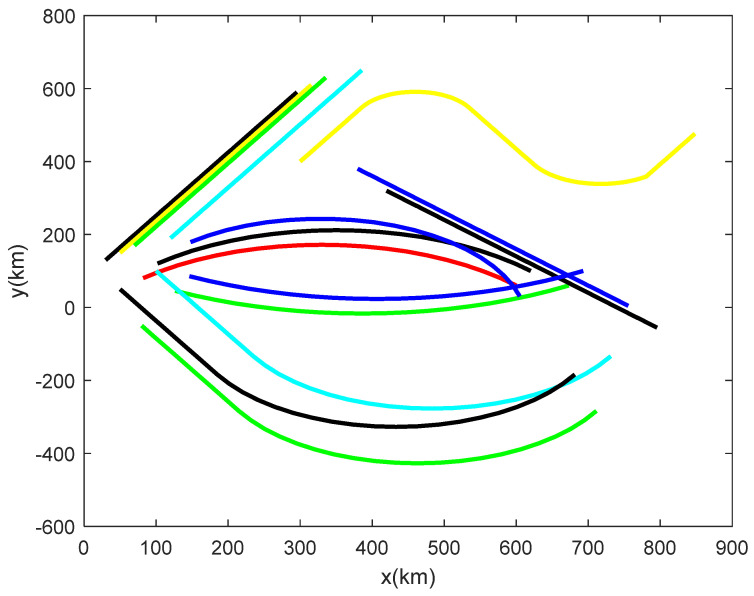
Two−dimensional coordinate system trajectory diagram.

Different motion models of maneuvering targets are simulated in this subsection, and the 15 trajectory samples shown in Figure 4 are used as the processing objects of the multi-feature fusion trajectory clustering algorithm. Among them, there are six curves of the uniform linear motion model, five curves of the coordinated turning motion model, and four curves of the mixture of a uniform straight line and a coordinated turning.

By using the single-feature trajectory clustering algorithm and the proposed multi-feature fusion-based trajectory clustering algorithm, respectively, the comparison results as shown in Table 1 are obtained.

As shown in Table 1, the mentioned 15 trajectory samples cannot be accurately classified by the single-feature trajectory clustering algorithm. However, the multi-feature fusion-based trajectory clustering algorithm can achieve the correct classification of the trajectory samples, which is beneficial for us in updating the state transition matrix. 

Comparing the proposed multi-feature fusion-based OPTICS algorithm with the common trajectory clustering algorithms, the clustering results are shown in Table 2. 

In Table 2, it is found that the recognition rate of the DBSCAN and DENCLUE algorithms is about 80%. The OPTICS algorithm is more accurate, and the recognition rate can reach about 90%. However, the multi-feature fusion-based OPTICS algorithm can improve the recognition accuracy more effectively, and the recognition rate can reach about 95%.

### 5.3. Passive Tracking of High-Maneuvering Targets

The STMU-EKF algorithm is simulated for high-maneuvering target tracking, compared with the EKF algorithm and the IMM-EKF algorithm. The state transition probability prior matrix of the IMM-EKF algorithm is [0.9 0.05 0.05]; that is, the initial probability of the uniform linear motion is 0.9, while the initial probability of the coordinated turning models is 0.05. 

The initial state transition matrix is divided into three types, which are the state transition matrix $F_{1}$ of the uniform linear motion, the state transition matrix $F_{2}$ of the uniform downward turning motion, and the state transition matrix $F_{3}$ of uniform upward turning motion, which are expressed as

(32) $$F_{1}=\left[\begin{array}{cccc} 1 & 1 & 0 & 0\\ 0 & 1 & 0 & 0\\ 0 & 0 & 1 & 1\\ 0 & 0 & 0 & 1 \end{array}\right],$$

(33) $$F_{2}=\left[\begin{array}{cccc} 1 & \sin\omega_{2}/\omega_{2} & 0 & \left(\cos\omega_{2}-1\right)/\omega_{2}\\ 0 & \cos\omega_{2} & 0 & -\sin\omega_{2}\\ 0 & \left(1-\cos\omega_{2}\right)/\omega_{2} & 1 & \sin\omega_{2}/\omega_{2}\\ 0 & \sin\omega_{2} & 0 & \cos\omega_{2} \end{array}\right],$$

(34) $$F_{3}=\left[\begin{array}{cccc} 1 & \sin\omega_{3}/\omega_{3} & 0 & \left(\cos\omega_{3}-1\right)/\omega_{3}\\ 0 & \cos\omega_{3} & 0 & -\sin\omega_{3}\\ 0 & \left(1-\cos\omega_{3}\right)/\omega_{3} & 1 & \sin\omega_{3}/\omega_{3}\\ 0 & \sin\omega_{3} & 0 & \cos\omega_{3} \end{array}\right],$$

where $\omega_{2}=\frac{\pi }{180}$ and $\omega_{3}=-\frac{\pi }{180}$.

**Figure 5:**
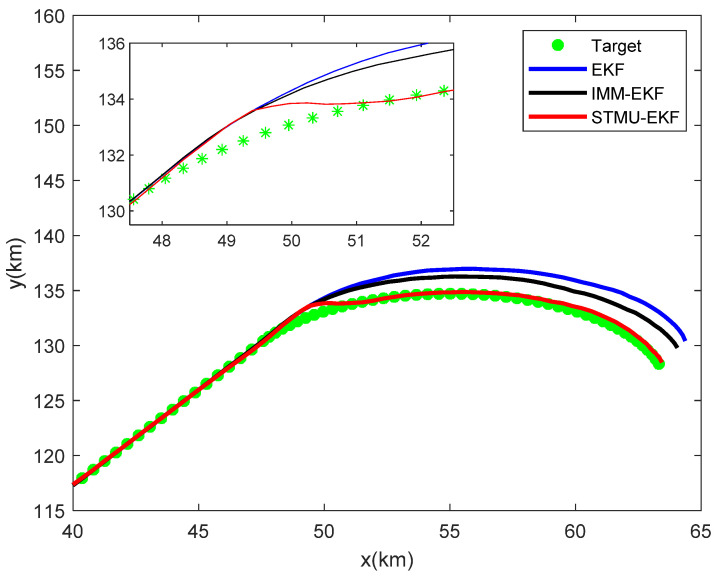
The target tracking trace of the three passive target tracking algorithms.

**Figure 6:**
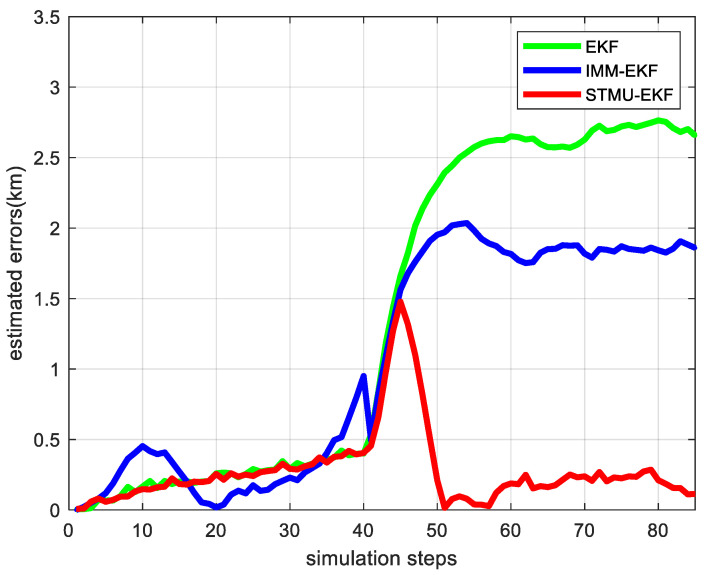
The target tracking error of the three passive target tracking algorithms.

Track the target within 85 sampling intervals, and the target tracking traces of the EKF algorithm, the IMM-EKF algorithm, and the STMU-EKF algorithm are shown in Figure 5. The target tracking errors of the EKF algorithm, the IMM-EKF algorithm, and the STMU-EKF algorithm are shown in Figure 6.

As shown in Figure 5 and Figure 6, at the first 40 sampling moments, the state transition matrix of EKF remains $F_{1}$ unchanged. Meanwhile, the tracking error of STMU-EKF is similar to that of EKF because the state transition matrix $F_{1}$ is not updated. As a multi-model algorithm, IMM-EKF is not completely derived from the single uniform linear motion model, so its error is the largest at the same time.

At the 41st sampling time, the target motion model is transformed from a uniform linear motion to a uniform downward turning motion. At this time, the EKF algorithm still keeps the original state transition matrix $F_{1}$ unchanged, which leads to the worst tracking results. The IMM-EKF algorithm performs multi-model adaptive adjustment due to the control of the state transition probability prior matrix, and its error is slightly smaller than that of EKF. Different from these two algorithms, the result of STMU-EKF is not ideal at the 41st~46th sampling moment, but the state transition matrix is updated at the 47th sampling moment. Substitute the updated state transition matrix $F_{2}$ into the algorithm for target tracking, and the error of the STMU-EKF algorithm gradually decreases. Finally, stabilizes within a certain error range and is much smaller than the other two algorithms. Therefore, the algorithm proposed in this paper is suitable for the high-maneuvering target tracking.

### 5.4. Multi-Sensor Collaborative Management

**Figure 7:**
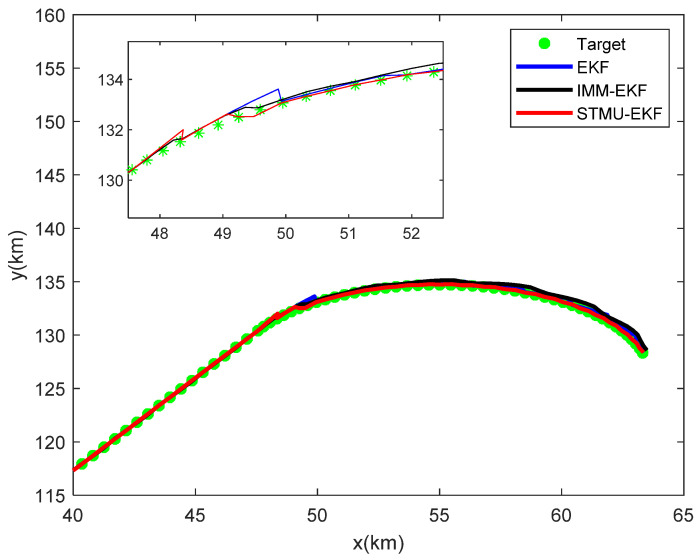
The target tracking trace in the multi-sensor collaborative management model.

**Figure 8:**
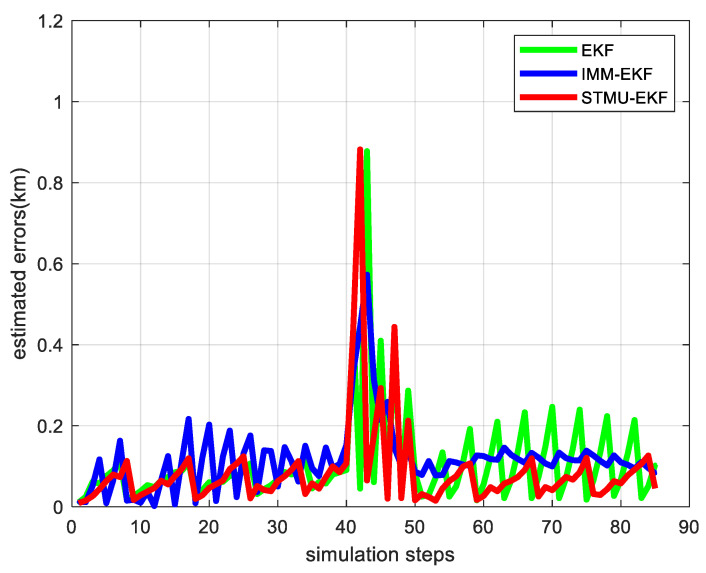
The target tracking error in the multi-sensor collaborative management model.

On the basis of passive tracking of high-maneuvering targets, radar is introduced for error correction; that is, the STMU-EKF algorithm is applied to the multi-sensor collaborative management model, and the target tracking trace in which the green points represent the true motion trajectory of the target is shown in Figure 7, and the target tracking error is shown in Figure 8.

In the first 40 sampling intervals, that is, when the tracking target is in a uniform linear motion model, the error values of the EKF algorithm and the STMU-EKF algorithm both fluctuate within a certain range, and their tracking performance is similar; However, due to the unknown state transition probability matrix, the error of the IMM-EKF algorithm is slightly larger than that of the EKF algorithm and the STMU-EKF algorithm, so the number of radar radiations is also slightly larger.

**Figure 9:**
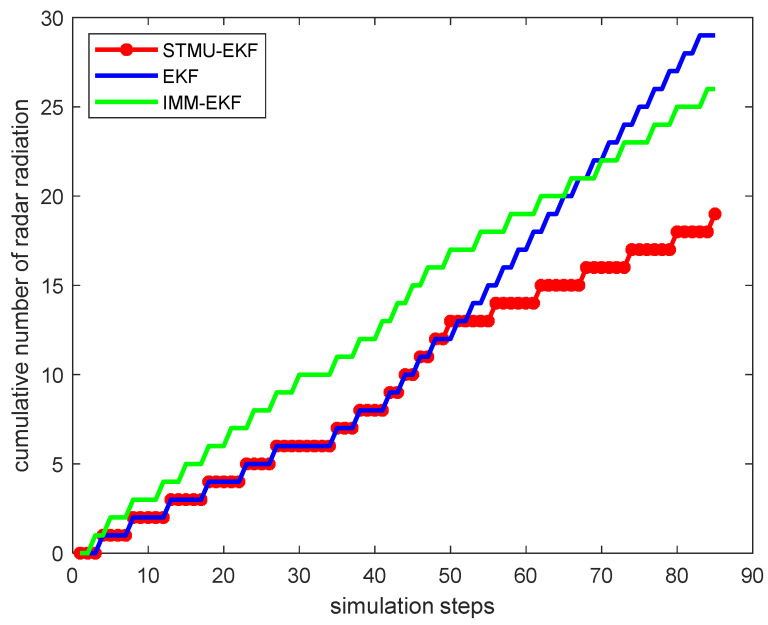
Radar radiation states of three algorithms.

**Figure 10:**
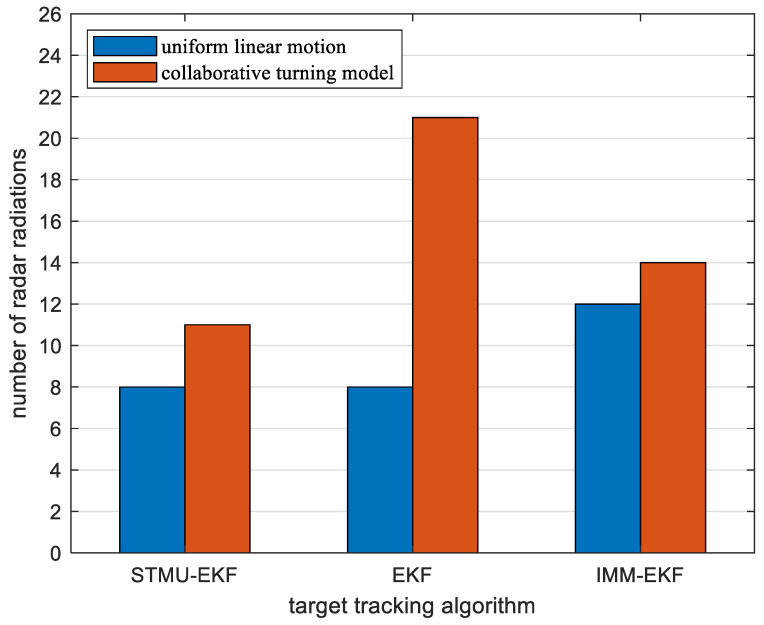
Radar radiation times of three algorithms in different motion states.

Starting from the 41st sampling interval, the target changes from a uniform linear motion model to a coordinated turning motion model. At this time, the original state transition matrix is no longer suitable for the current target motion model, so the traditional EKF algorithm has a large error. At the same time, because the IMM-EKF algorithm can convert between models, the error is slightly smaller than that of the EKF algorithm, and its radar radiates 14 times. The STMU-EKF algorithm starts to update the state transition matrix at the 41st sampling interval and completes the update at the 46th sampling interval. The STMU-EKF algorithm that obtains the correct state transition matrix improves the target tracking accuracy and reduces the number of radar radiations. It can be seen from Figure 7 and Figure 8 that the error of the STMU-EKF algorithm gradually decreases from the 46th sampling interval, and its predicted trajectory gradually approaches the real trajectory; It can be seen from Figure 9 and Figure 10 that when using the STMU-EKF algorithm, the radar radiates 11 times in the last 45 simulation intervals, which is lower than that using IMM-EKF algorithm and EKF algorithm. From the perspective of the whole sampling process, the radar radiates 19 times when using STMU-EKF, which is much lower than the 29 times when using the EKF algorithm and 27 times when using IMM-EKF. Therefore, the multi-sensor collaborative management model based on trajectory clustering designed in this paper can improve the radar tracking accuracy and reduce the number of radar radiation, thereby achieving LPI tracking.

### 5.5. Performance Analysis

For the multi-sensor collaborative management model based on trajectory clustering, different accumulated error thresholds are selected to simulate the high-maneuvering target tracking, and the number of radar radiations and mean estimation error are counted, as shown in Table 3.

As shown in Table 3, a small threshold can decrease the average estimation error, but it also increases the number of radar radiation, which is not conducive to the radar’s LPI tracking. Too large a threshold is not suitable either, which leads to a large average estimation error though the number of radar radiation is reduced. Therefore, it is best to set the threshold value around 0.1 when tracking the high-maneuvering targets.

In addition, when the radar threshold is 0.1, this paper also selects different fluctuation parameter thresholds for tracking simulation and counts their radiation times and mean estimation errors, as shown in Table 4.

It can be seen from Table 4 that the setting of the fluctuation parameter threshold also has a great influence on the radar radiation and the average estimation error. When the threshold is too large, the model cannot update the state transition matrix in time, the number of radar radiation is large, and the average estimation error is large; If the threshold is too small, too many times of trajectory clustering are required, and each clustering requires 5~6 simulation intervals, which will cause the phenomenon of trajectory clustering blockage, which will increase the number of radar radiation and the average estimation error. Therefore, it is best to set the threshold around 0.5.

## 6. Conclusions

In this paper, the STMU-EKF algorithm is firstly proposed to solve the problem of low tracking accuracy caused by the unknown motion model of high-maneuvering targets. In the case of a large target tracking error, STMU-EKF uses trajectory clustering to realize target motion state estimation and updates the state transition matrix according to the estimated value of the motion model and the observation value of the multi-station passive sensor, which solves the problem of poor tracking accuracy of traditional passive tracking algorithm in high-maneuvering target tracking. The trajectory clustering algorithm uses four spatial features of inter-frame trajectory mean M, trajectory angle $\theta_{k}$, instantaneous speed $v_{k}$ and average speed $\bar{v}$ as eigenvalues for fusion clustering to improve the clustering accuracy. On this basis, considering that only using passive sensors for target tracking cannot often meet the requirements of high target tracking accuracy, this paper introduces active radar for indirect radiation and proposes a multi-sensor collaborative management model based on trajectory clustering. This model optimally allocates the sensor by judging the accumulated errors of the eigenvalue of the error covariance matrix, that is, the accumulated value of the difference of the prediction covariance matrix trace in the adjacent interval. This model also makes the decision to update the state transition matrix according to the fluctuation parameters, that is, the difference between the prediction value and the observation value of the filtering algorithm in the adjacent intervals. In this paper, the target tracking simulation of the multi-station passive sensor for high-maneuvering targets is carried out, the target tracking error and radar radiation times based on multi-sensor cooperative management are analyzed, and the setting of the accumulated errors of the eigenvalue of the error covariance matrix and fluctuation parameter threshold is discussed. The simulation results show that the STMU-EKF algorithm designed in this paper can significantly improve the tracking accuracy of passive sensors for high-maneuvering targets, and the multi-sensor collaborative management model based on trajectory clustering can further improve the tracking accuracy of high-maneuvering targets while ensuring the low radiation times of radar. The research conclusion is helpful to improve the concealment of the fighter and realize the LPI tracking. In the future, low intercept probability tracking methods for multiple high-maneuvering targets can be studied. In addition, the radar networking technology can be applied to the sensor management model proposed in this paper to further improve the LPI performance.

## Figures and Tables

**Table 1:** Two-dimensional coordinate system trajectory clustering results.

	Uniform Linear Motion (6 Curves in All)	Coordinated Turning (5 Curves in All)	Linear Motion+ Coordinated Turning (4 Curves in All)
Trajectory mean	83.3%	83.3%	100%
Trajectory length	100%	83.3%	75%
Trajectory angle	50%	40%	50%
Instantaneous speed	83.3%	100%	75%
Average speed	100%	80%	75%
Proposed multi-feature fusion-based OPTICS	100%	100%	100%

**Table 2:** The comparison of trajectory clustering algorithms.

	Uniform Linear Motion	Coordinated Turning	Linear Motion + Coordinated Turning
DBSCAN	78%	73%	69%
DENCLUE	83%	80%	74%
OPTICS	92%	90%	87%
Proposed multi-feature fusion-based OPTICS	98%	94%	92%

**Table 3:** The comparison of accumulated error thresholds.

Accumulated error threshold	10.0	5.0	0.5	0.1	0.04
Number of radar radiations	5	8	13	18	27
Mean estimation error/km	1.3	0.83	0.21	0.073	0.048

**Table 4:** The comparison of fluctuation parameter thresholds.

Fluctuation parameter threshold	7.0	4.0	2.0	0.5	0.1
Number of radar radiations	10	13	16	18	20
Mean estimation error/km	1.2	0.43	0.122	0.073	0.052

## Abbreviations

The following abbreviations are used in this manuscript:

LPI	Low Probability Of Intercept
EKF	Extended Kalman Filter
STMU-EKF	State Transition Matrix Update-Based Extended Kalman Filter
MIMO	Multiple-Input Multiple-Output
DBSCA	Density-Based Applied Spatial Clustering Algorithm With Noise
AIS	Automatic Identification System
DENCLUE	Density-Based Clustering
OPTICS	Ordering Points To Identify The Clustering Structure
IMM-EKF	Interacting Multiple Model-Based Extended Kalman Filter
DTOA	Difference Time Of Arrival
